# Pregnancy and neonatal outcomes of ICSI using pentoxifylline to identify viable spermatozoa in patients with frozen-thawed testicular spermatozoa

**DOI:** 10.3389/fendo.2024.1364285

**Published:** 2024-05-15

**Authors:** Jing Dong, Mingru Yin, Ling Wu, Tiantian Wang, Menghui Li, Wei Zhang, Meng Ma, Bin Li

**Affiliations:** Department of Assisted Reproduction, the Ninth People’s Hospital affiliated to Shanghai Jiaotong University School of Medicine, Shanghai, China

**Keywords:** pentoxifylline, birth defect, testicular sperm, ICSI, propensity score matching

## Abstract

**Introduction:**

Although the effectiveness of pentoxifylline (PF) as a selective inhibitor of phosphodiesterase to enhance sperm motility through increasing cyclic nucleotide in cases of absolute asthenozoospermia has been demonstrated for ICSI, data related to babies born from the PF-ICSI are still severely lacking. Concerns have been raised regarding the potential embryotoxicity of PF due to the controversial results obtained from the analysis of this compound on animal embryo development. This study aimed to determine whether the application of PF to trigger frozen-thawed TESA (testicular sperm aspiration) spermatozoa increases the risk of adverse obstetric and neonatal outcomes compared with non-PF frozen-thawed TESA ICSI and conventional ICSI using fresh ejaculation.

**Materials and methods:**

A total of 5438 patients were analyzed in this study, including 240 patients underwent PF-TESA ICSI (ICSI using PF triggered frozen-thawed testicular spermatozoa), 101 patients underwent non-PF TESA ICSI (ICSI using frozen-thawed testicular spermatozoa) and 5097 patients underwent conventional ICSI using fresh ejaculation. Propensity score matching was executed to control the various characteristics of patients.

**Results:**

No significant differences in pregnancy outcomes were observed among the three groups (PF-TESA ICSI, non-PF TESA ICSI and conventional ICSI), including biochemical pregnancy, clinical pregnancy, implantation, miscarriage, ectopic pregnancy, multiple pregnancy, and live birth, following propensity score matching. Additionally, neonatal outcomes were found to be similar among the three groups, with no statistical differences observed in the birth defect, birth weight, gestational age, preterm birth, and early-neonatal death.

**Discussion and conclusion:**

PF-ICSI may be an alternative treatment in patients using frozen-thawed testicular spermatozoa, resulting in comparable pregnancy and neonatal outcomes.

## 1 Introduction

Infertility affects approximately 14% of fertility couples with the male partner accounting for about 37% of all cases (1). In male partners evaluated for infertility, azoospermia accounts for approximately 5%–10% (2). Successful application of intracytoplasmic sperm injection (ICSI) in humans (3) enables couples with severe male factors (such as azoospermia) to receive ICSI treatment (4). However, testicular sperm is usually immotile or shows only a minor twitching movement upon extraction (5). Several studies have reported that although immotile testicular sperm can be utilized for oocyte fertilization, it has been associated with lower quality embryos and decreased pregnancy rates (6, 7). Previous studies have shown that the use of human immotile spermatozoa for ICSI has an adverse effect on fertilization and pregnancy rates (8–10).

In order to improve the ICSI outcomes, pentoxifylline (PF) has been explored as a potential treatment approach for male infertility (11, 12). PF, a member of the phosphodiesterase inhibitors family, acts by elevating the levels of cyclic nucleotide such as cyclic adenosine monophosphate and cyclic guanosine monophosphate (13, 14). It activates sperm motility through the cyclic adenosine monophosphate pathway, thereby inducing downstream sperm tail protein phosphorylation (15). The efficacy of PF, as a selective inhibitor of phosphodiesterase that can activate immotile testicular sperm, has been showed in previous studies (16–19).

However, serious concerns have arisen about the potential embryo toxicity of PF due to the controversial results obtained from analyzing its effects on animal embryo development (20, 21), and there is a severe lack of data related to babies born from the PF-ICSI. In 2017, Navas et al. reported that ICSI using spermatozoa exposed with PF did not enhance adverse obstetric and neonatal outcomes, including malformation rates (22). However, the study was highly heterogeneous regarding sperm source (epididymal and testicular) and sperm type (fresh and frozen-thawed). In addition, the study cohort was small, and it lacked an adequate control group. Considering the emerging concerns about potential adverse consequences of PF, it is timely to investigate the available information on the use of PF in the treatment of male factor infertility, and this study will compare pregnancy and neonatal outcomes, including congenital malformations among the PF-TESA ICSI, non-PF TESA ICSI and non-PF conventional ICSI using fresh ejaculation, which will provide some pointers to its future use.

## 2 Materials and methods

### 2.1 Study setting and patients

This was a retrospective cohort study using the data collected from the Department of Assisted Reproduction of the Ninth People’s Hospital of Shanghai Jiaotong University School of Medicine, from April 2013 to June 2021. This study aimed to investigate the pregnancy and neonatal outcomes of infertile couples who underwent PF-TESA ICSI treatments, with non-PF TESA ICSI and non-PF conventional ICSI using fresh ejaculation as the control groups, respectively.

The following cycles were excluded: (1) oocyte donation cycles; (2) deformed uterus; (3) repeated FET cycles of a patient; (4) with a history of genetic diseases or chromosomal disorders; (5) cycles lost to follow up. In this study, we analyzed 240 FET cycles involving PF-TESA ICSI (study group), and compared them to the non-PF TESA ICSI (control group 1) and non-PF conventional ICSI (control group 2) groups consisting of 5198 FET cycles. Only patients with obstructive azoospermia were included in the study. To eliminate bias between groups, propensity scores were calculated using logistic regression model in SPSS version 26.0 (23), and matching was performed, without replacement using propensity scores with the nearest neighbor, largest matching algorithm. Based on the size of the sample, patients in the conventional ICSI group were matched in the 1:3 ratio, while those in the non-PF TESA ICSI group were matched 1:1. Matching was based on female and male age, female and male body mass index, duration of infertility, gravidity, parity, number of previous cycles with no available embryos, number of previous transfer failure cycles, type of endometrial preparation, endometrial thickness on embryo transfer day, number of embryos per transfer, and causes of infertility.

**Figure 1:**
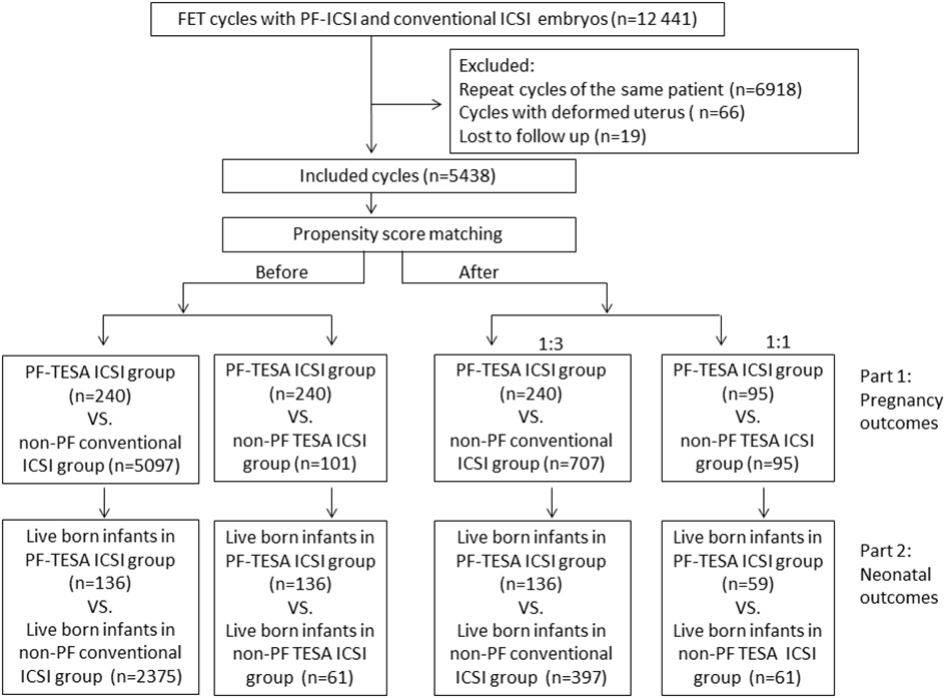
The flowchart of the study. FET, frozen–thawed embryo transfer; ICSI, intracytoplasmic sperm injection; PF-TESA ICSI, ICSI using PF triggered frozen-thawed testicular spermatozoa; non-PF TESA ICSI, ICSI using frozen-thawed testicular spermatozoa; non-PF conventional ICSI, ICSI using fresh ejaculation.

This study consisted of two parts: we firstly compared pregnancy outcomes among three different groups: PF-TESA ICSI, non-PF TESA ICSI and non-PF conventional ICSI; whereafter, we further analyzed neonatal outcomes in these three groups (**Figure 1**).

The study was conducted in accordance with the Declaration of Helsinki and approved by the Ethics Committee of Shanghai Ninth People’s Hospital, which is affiliated to Shanghai Jiao Tong University School of Medicine. Informed consents were obtained from all participants after they received counseling on infertility treatments.

### 2.2 Treatment

The procedures of ovarian stimulation, ICSI, embryo culture, cryopreservation and thawing, endometrial preparation, and FET were performed using a standardized method, as previously described (24, 25).

The testicular spermatozoa sample was derived from testicular sperm aspiration (TESA). Testicular spermatozoa samples were cryopreserved, prior to commencing ovarian stimulation. The frozen-thawed procedure for TESA samples was as follows: Mixed the testicular tissue suspension containing sperm in a one-to-one ratio with cryopreservation medium (Origio, Denmark) incrementally. After each addition, ensured thorough blending of the solution and allowed it to equilibrate at room temperature for 10 minutes. Created an incision on one side of the sperm straw (CBS, France) and introduced the combined sample into this opening (approximately 50 µl per straw). And the cooling process should be executed as follows: Gently lowered the sperm straw from a height of around 50cm above the liquid nitrogen surface within 1 minute until it hovered just above the liquid nitrogen, and then maintained this position until the sperm solution solidified, before submerging it directly into liquid nitrogen (-196°C). On the day of oocyte pick up, took out the sperm straw from liquid nitrogen and immersed the sperm-containing end into preheated oil at 37°C promptly until the sperm sample was completely dissolved, then transferred it into the sperm droplet in an ICSI dish for subsequent ICSI procedures.

In the study group, ICSI was performed after exposing spermatozoa to commercial PF reagent (GaoHong biotechnology co., China). Briefly, about 20μl ready-to-use PF solution was directly added to the sperm droplet in an ICSI dish, cultured at 37°C for around five minutes, subsequently motile spermatozoa were chosen and transferred to PVP droplets and mechanically washed by repeatedly blowing and sucking at different parts of the polyvinylpyrrolidone droplet using an injection pipette, and the ICSI procedure was performed in the classical manner.

In the non-PF thawed TESA ICSI group, only motile spermatozoa were selected for insemination, too. In instances where no motile spermatozoa were identified, alternative options such as fresh testicular puncture or egg freezing were considered, and these cases would be excluded from the study.

Fertilization assessment was conducted about 16-18 h after ICSI, with successful fertilization being characterized by the presence of two distinct pronuclei. The embryos were evaluated on the third day after fertilization based on ASEBIR embryo assessment criteria (26), and the Gardner and Schoolcraft system (27) was used to grade blastocysts. Cleavage embryos that scored Grades I and II, regarded as good-quality embryos, were cryopreserved, while poor-quality embryos (Grades III or IV) were subjected to extended culture, and blastocysts that scored equal or better than 3BB on day 5 or 4CC on day 6 were defined as available blastocysts and frozen.

The procedures of vitrification of thawing were performed as described in our previous study (28). Briefly, embryos underwent an initial incubation period for 10-15min in an equilibration solution, followed by a brief immersion of approximately 60s in a vitrification solution. Subsequently, the embryos were carefully loaded on the Cryotop strip in a minimal volume and promptly submerged into liquid nitrogen. For the thawing process, the Cryotop strip was immersed in a 37°C thawing solution for around 60s, after which the embryos were transferred into a diluent solution for 3 min and then washed twice in a wash solution for 5 min, respectively, at room temperature. Finally, the embryos were incubated in a culture medium supplemented with 10% serum substitute supplements (Irvine Scientifc) at 37°C, CO_2_ 6%.

### 2.3 Outcome assessment

The fertility outcome terms in this study were defined as previously described (29). The serum human chorionic gonadotropin (hCG) concentration of the patients was assessed on the 12th day following FET. Biochemical pregnancy was identified by a positive hCG test. Clinical pregnancy was confirmed by the presence of a gestational sac during ultrasound examination on the 35th day after embryo transfer. Miscarriage was characterized by the loss of an intrauterine pregnancy before the 24th week of gestation. Ectopic pregnancy was described as a pregnancy occurring outside the uterine cavity. Live birth was defined as the delivery of a living baby after 24 weeks of gestation. Gestational age was calculated by adding two weeks to the number of weeks since fertilization. Preterm delivery was defined as a gestational age of less than 37 weeks, while low birth weight was defined as a birth weight of less than 2500 g. Early neonatal death was defined as the death of a live-born baby within seven days.

Assessment for birth defects in this study was described in previous reports (30). Couples completed a telephonic interview during each stage of pregnancy, providing data on gestational weeks, pregnancy complications, infant gender, neonatal birth weight and diseases, and any live birth defects, et al. When attempts to contact couples failed, the information on the questionnaire was obtained through family planning service agencies. Positive cases of birth defects and neonatal death were confirmed by medical records. A specially designated nurse reviewed postpartum neonates with birth defects to ensure that they met the case definition of the Chinese Birth Defects Monitoring Program. Based on the International Classification of Diseases, 10th Revision, birth defects were classified and coded.

### 2.4 Statistical analyses

All statistical analyses were conducted by SPSS version 26.0 software (SPSS Inc., Chicago, IL, USA). A normality test was performed on all continuous variables. Means ± standard deviation were used for presentation, and the t-test was employed to assess the differences for data that followed a normal distribution. Alternatively, data were presented as medians (ranges) and analyzed using the Mann-Whitney test. Qualitative data were presented as percentages and statistical differences were assessed using the Chi-squared test or Fisher’s exact test when appropriate. Odds ratios (OR) was calculated when assessing the pregnancy and neonatal outcomes of the patients. Results were reported as odds ratio (ORs) with 95% confidence intervals (CIs). Value of *P* < 0.05 was considered statistically significant.

## 3 Results

A total of 5438 FET cycles were analyzed in this study, including 240 cycles in the PF-TESA ICSI group, 101 cycles in the non-PF TESA ICSI group, and 5097 cycles in the conventional ICSI group. In comparison of characteristics observed between the PF-TESA and non-PF TESA ICSI groups, the age of both female and male patients was significantly higher in the non-PF TESA ICSI group (37(30-58) vs 34(23-51) and 39(31-64) vs 36(25-65), respectively, *P*<0.01). Additionally, the number of previous cycles with no available embryos, the number of embryo transfer, as well as the type of embryo transferred also illustrated significant disparities between the two groups (*P*<0.05). In comparison of PF-TESA ICSI and conventional ICSI, the ages were found to be significantly higher in the conventional ICSI group (36(23-57) vs 34(23-51) and 38(24-70) vs 36(25-65), respectively, *P*<0.01), while other parameters, such as female body mass index, gravidity, number of previous transfer failure cycles, number of previous cycles with no available embryos, causes of infertility, number of embryo transfer, endometrial thickness and preparation protocol were also markedly different between the groups (*P*<0.05). As differences in patients’ characteristics could have potential effects on fertility outcomes, propensity score matching was employed to control for the divergent characteristics of patients. After matching, patients’ characteristics among the three groups were found to be similar (*P*>0.05), with the baseline patient characteristics before and after the propensity score matching summarized in **Table 1**.

**Table 1:** Baseline characteristics of patients among the PF-TESA ICSI, non-PF TESA ICSI and non-PF conventional ICSI groups.

Characteristics	PF-TESA ICSI Study group	PF-TESA ICSI vs. non-PF conventional ICSI				PF-TESA ICSI vs. non-PF TESA ICSI				
		Before matching		After matching		Before matching		After matching		
		Control group 2	*P* value	Control group 2	*P* value	Control group 1	*P* value	Study group	Control group 1	*P* value
No. of patients	240	5097		707		101		95	95	
Female age, years	34(23-51)	36(23-57)	**<0.001**	34(23-53)	0.861	37(30-58)	**<0.001**	36(27-49)	37(30-52)	0.331
Male age, years	36(25-65)	38(24-70)	**<0.001**	36(24-70)	0.888	39(31-64)	**<0.001**	37(28-65)	38(31-62)	0.210
Female body mass index, kg/m^2^	21.23(15.63-33.62)	21.63(14.53-46.88)	**0.029**	21.22(15.05-39.06)	0.809	21.08(15.82-30.11)	0.774	21.26(16.73-32.39)	21.08(15.82-30.11)	0.642
Male body mass index, kg/m^2^	24.22(16.67-34.66)	24.22(16.14-54.35)	0.399	24.22(16.41-39.14)	0.589	24.14(15.36-35.49)	0.696	24.03(17.30-34.66)	24.14(15.36-35.49)	0.614
Duration of infertility, years	3(1-18)	3(1-23)	**0.001**	3(1-19)	0.854	3(0.5-17)	0.458	3(1-15)	3(0.5-14)	0.705
Gravidity	0(0-5)	0(0-9)	**<0.001**	0(0-6)	0.767	0(0-5)	0.081	0(0-3)	0(0-3)	0.204
Parity	0(0-2)	0(0-3)	0.201	0(0-2)	0.580	0(0-1)	0.795	0(0-2)	0(0-1)	0.436
No. of previous full-term births	0(0-2)	0(0-3)	0.225	0(0-2)	0.743	0(0-1)	0.803	0(0-2)	0(0-1)	0.589
No. of previous transfer failure cycles	0(0-4)	0(0-11)	**<0.001**	0(0-4)	0.233	0(0-4)	0.294	0(0-4)	0(0-4)	0.602
No. of previous no available embryo cycles	0(0-2)	0(0-8)	**0.001**	0(0-4)	0.875	0(0-2)	**0.002**	0(0-2)	0(0-2)	0.793
Causes of infertility										
Tubal factor infertility, n (%)	32(13.33)	2446(47.99)	**<0.001**	103(14.57)	0.636	14(13.86)	0.896	12(12.63)	11(11.58)	0.824
Polycystic ovary syndrome, n (%)	10(4.17)	447(8.77)	**0.013**	36(5.09)	0.565	2(1.98)	0.521	3(3.16)	2(2.11)	1.000
Endometriosis, n (%)	2(0.83)	454(8.91)	**<0.001**	4(0.57)	0.647	4(3.96)	0.066	1(1.05)	4(4.21)	0.368
Male factor infertility, n (%)	240(100.00)	2220(43.56)	**<0.001**	707(100.00)	–	101(100.00)	–	95(100.00)	95(100.00)	–
No. of embryos transferred	2(1-2)	2(1-2)	**0.016**	2(1-2)	0.802	2(1-2)	**<0.001**	2(1-2)	2(1-2)	1.000
Type of embryo transferred cycles			0.170		0.832		**0.004**			0.774
Cleavage embryo, n (%)	197(82.09)	4348(85.31)		576(81.47)		95(94.06)		88(92.63)	89(93.68)	
Blastocyst, n (%)	43(17.92)	749(14.69)		131(18.53)		6(5.94)		7(7.37)	6(6.32)	
Endometrial thickness, mm	10.80(5.10-22.80)	10.50(3.10-22.80)	**0.041**	10.80(5.70-18.90)	0.944	10.70(6.80-21.70)	0.404	10.50(5.10-17.30)	10.70(6.80-21.70)	0.898
Endometrium preparation			**0.042**		0.932		0.193			0.536
Natural cycles, n (%)	50(20.83)	905(17.76)		150(21.22)		26(25.74)		23(24.21)	23(24.21)	
Stimulated cycles, n (%)	142(59.17)	2806(55.05)		409(57.85)		49(48.51)		55(57.89)	49(51.58)	
Hormone therapy cycles, n (%)	48(20.00)	1386(27.19)		148(20.93)		26(25.74)		17(17.89)	23(24.21)	

PF-TESA ICSI, ICSI using PF triggered frozen-thawed testicular spermatozoa; non-PF TESA ICSI, ICSI using frozen-thawed testicular spermatozoa; non-PF conventional ICSI, ICSI using fresh ejaculation. Bold indicates *P* < 0.05.

Pregnancy outcomes were evaluated before and after matching and summarized in **Tables 2**, **3**. Our results demonstrated no significant differences between PF-TESA ICSI and non-PF TESA ICSI, before and after propensity score matching (*P*>0.05). When compared to conventional ICSI, no significant differences were found in the rates of ectopic pregnancy, multiple gestation, miscarriage, multiple birth, preterm delivery and the average of gestational age (*P*>0.05). However, we found that the rates of biochemical pregnancy, clinical pregnancy, intrauterine implantation, and live birth were significantly higher in the PF-TESA ICSI group than those in the conventional ICSI group (57.92% vs 50.42%, 54.17% vs 46.34%, 40.10% vs 32.78%, 47.08% vs 38.41%, all *P*<0.05, respectively). In an effort to control effects of patients’ different characteristics on pregnancy outcomes, we employed propensity score matching. After matching, no significant differences were found with regards of pregnancy outcomes between the two groups.

**Table 2:** Pregnancy outcomes of patients between the PF-TESA ICSI and non-PF TESA ICSI groups.

Outcomes	PF-TESA ICSI (study group) vs. non-PF TESA ICSI (control group 1)							
	Before matching				After matching			
	Study group	Control group 1	*P* value	OR(95%CI)	Study group	Control group 1	*P* value	OR(95%CI)
Biochemical pregnancy	57.92(139/240)	58.42(59/101)	0.932	0.980(0.611-1.570)	57.89(55/95)	61.05(58/95)	0.658	0.877(0.491-1.566)
Clinical pregnancy	54.17(130/240)	53.47(54/101)	0.906	1.029(0.645-1.640)	53.68(51/95)	56.84(54/95)	0.662	0.880(0.497-1.560)
Intrauterine implantation	40.10(158/394)	35.53(70/197)	0.282	1.215(0.852-1.732)	34.05(63/185)	37.84(70/185)	0.448	0.848(0.555-1.298)
Ectopic pregnancy	0.77(1/130)	3.70(2/54)	0.206	0.202(0.018-2.271)	1.96(1/51)	3.70(2/54)	0.592	0.520(0.046-5.916)
Multiple gestation	19.23(25/130)	27.78(15/54)	0.201	0.619(0.296-1.295)	23.53(12/51)	27.78(15/54)	0.619	0.800(0.332-1.927)
Miscarriage	12.31(16/130)	5.56(3/54)	0.171	2.386(0.666-8.552)	5.88(3/51)	5.56(3/54)	1.000	1.063(0.204-5.522)
Live birth	47.08(113/240)	48.51(49/101)	0.809	0.944(0.593-1.504)	49.47(47/95)	51.58(49/95)	0.772	0.919(0.520-1.624)
Multiple birth	20.35(23/113)	24.49(12/49)	0.557	0.788(0.355-1.747)	25.53(12/47)	24.49(12/49)	0.906	1.057(0.420-2.663)
Gestational age (weeks,mean (SD))	39(26-42)	38(24-41)	0.604	–	38(34-41)	38(24-41)	0.967	–
Gestational age (weeks,n(%))			0.256	–			0.544	–
< 32	1(0.88)	1(2.04)			0(0.00)	1(2.04)		
32 - 36	10(8.85)	9(18.37)			5(10.64)	9(18.37)		
37 - 40	95(84.07)	36(73.47)			40(85.11)	36(73.47)		
> 42	7(6.19)	3(6.12)			2(4.26)	3(6.12)		
Preterm delivery (<37 weeks)(%)	9.73(11/113)	20.41(10/49)	0.063	0.421(0.166-1.069)	10.64(5/47)	20.41(10/49)	0.188	0.464(0.146-1.479)

PF-TESA ICSI, ICSI using PF triggered frozen-thawed testicular spermatozoa; non-PF TESA ICSI, ICSI using frozen-thawed testicular spermatozoa.

**Table 3:** Pregnancy outcomes of patients between the PF-TESA ICSI and non-PF conventional ICSI groups.

Outcomes	PF-TESA ICSI (study group) versus non-PF conventional ICSI (control group 2)						
	Study group	Before matching			After matching		
		Control group 2	*P* value	OR(95%CI)	Control group 2	*P* value	OR(95%CI)
Biochemical pregnancy	57.92(139/240)	50.42(2570/5097)	**0.023**	**1.353(1.041-1.759)**	60.25(426/707)	0.524	0.908(0.674-1.222)
Clinical pregnancy	54.17(130/240)	46.34(2362/5097)	**0.018**	**1.368(1.055-1.774)**	56.15(397/707)	0.593	0.923(0.688-1.238)
Intrauterine implantation	40.10(158/394)	32.78(2863/8734)	**0.003**	**1.373(1.117-1.688)**	40.96(478/1167)	0.764	0.965(0.765-1.218)
Ectopic pregnancy	0.77(1/130)	1.74(41/2362)	0.405	0.439(0.060-3.216)	0.50(2/397)	0.573	1.531(0.138-17.024)
Multiple gestation	19.23(25/130)	19.90(470/2362)	0.853	0.958(0.613-1.500)	17.38(69/397)	0.632	1.132(0.681-1.880)
Miscarriage	12.31(16/130)	15.54(367/2362)	0.320	0.763(0.447-1.303)	14.86(59/397)	0.469	0.804(0.445-1.453)
Live birth	47.08(113/240)	38.41(1959/5097)	**0.007**	**1.425(1.099-1.848)**	47.52(336/707)	0.906	0.982(0.733-1.317)
Multiple birth	20.35(23/113)	21.34(418/1959)	0.804	0.942(0.589-1.508)	18.45(62/336)	0.655	1.129(0.662-1.927)
Gestational age (weeks)	39(26-42)	38(25-42)	0.241	–	39(31-41)	0.755	–
Gestational age (weeks, n(%))			0.172	–		0.778	–
< 32	1(0.88)	36(1.84)			2(0.60)		
32 - 36	10(8.85)	257(13.12)			36(10.71)		
37 - 40	95(84.07)	1606(81.98)			284(84.52)		
> 42	7(6.19)	60(3.06)			14(4.17)		
Preterm delivery (<37 weeks)(%)	9.73(11/113)	14.55(285/1959)	0.155	0.633(0.336-1.195)	11.31(38/336)	0.642	0.846(0.417-1.716)

PF-TESA ICSI, ICSI using PF. triggered frozen-thawed testicular spermatozoa; non-PF conventional ICSI, ICSI using fresh ejaculation without PF trigger. Bold indicates *P* < 0.05.

To further assess the clinical safety of PF treatment, we analyzed the neonatal outcomes of patients who underwent PF-TESA ICSI (**Tables 4**, **5**). A total of 2572 live-born infants were examined, including 136 newborns from PF-TESA ICSI group, 61 from non-PF TESA ICSI group and 2375 from the conventional ICSI group. Our results demonstrated that no significant differences existed across the three groups in terms of birth weight and low birth weight (*P*>0.05). Early neonatal death was not observed in the PF-TESA and non-PF TESA ICSI groups, though four cases were recorded in the conventional ICSI group, without any significant differences (*P*>0.05). After matching, no statistically significant differences in neonatal outcomes were found among the three groups, which was consistent with the results before matching.

**Table 4:** Neonatal outcomes of patients between the PF-TESA ICSI and non-PF TESA ICSI groups.

Outcomes	PF-TESA ICSI (study group) vs. non-PF TESA ICSI (control group 1)							
	Before matching				After matching			
	Study group	Control group 1	*P* value	OR(95%CI)	Study group	Control group 1	*P* value	OR(95%CI)
Live born infants (*n*)	136	61	0.454	0.788(0.422-1.471)	59	61	0.881	1.057(0.509-2.195)
Single	90(66.18)	37(60.66)			35(59.32)	37(60.66)		
Twins	46(33.82)	24(39.34)			24(40.68)	24(39.34)		
Birthweight (g)	3035.92 ± 592.51	3076.31 ± 506.69	0.645	–	2991.36 ± 553.62	3076.31 ± 506.69	0.382	–
Birthweight, (g, n(%))			0.474	–			0.543	–
< 1500 g	2(1.47)	0(0.00)			0(0.00)	0(0.00)		
1500–2499 g	15(11.03)	7(11.48)			9(15.25)	7(11.48)		
2500-4500 g	119(87.50)	54(88.52)			50(84.75)	54(88.52)		
> 4500 g	0(0.00)	0(0.00)			0(0.00)	0(0.00)		
Low birth weight, n(%)	17(12.50)	7(11.48)	0.839	1.102(0.432-2.813)	9(15.25)	7(11.48)	0.543	1.389(0.481-4.008)
Early neonatal death, n(%)	0(0.00)	0(0.00)	–	–	0(0.00)	0(0.00)	–	–
Congenital malformations, n(%)	3(2.21)	2(3.28)	0.646	0.665(0.108-4.087)	1(1.69)	2(3.28)	1.000	0.509(0.045-5.764)
Singletons	3	1			1	1		
Multiples	0	1			0	1		

PF-TESA ICSI, ICSI using PF triggered frozen-thawed testicular spermatozoa; non-PF TESA ICSI, ICSI using frozen-thawed testicular spermatozoa.

**Table 5:** Neonatal outcomes of patients between the PF-TESA ICSI and non-PF conventional ICSI groups.

Outcomes	PF-TESA ICSI (study group) versus non-PF conventional ICSI (control group 2)						
	Study group	Before matching			After matching		
		Control group 2	*P* value	OR(95%CI)	Control group 2	*P* value	OR(95%CI)
Live born infants (*n*)	136	2375	0.759	0.944(0.656-1.361)	397	0.539	1.139(0.753-1.723)
Single	90(66.18)	1541(64.88)			274(69.02)		
Twins	46(33.82)	834(35.12)			123(30.98)		
Birthweight (g)	3100(950-4450)	3010(690-5300)	0.821	–	3100(1450-4800)	0.368	–
Birthweight, (g, n(%))			0.257	–		0.126	–
< 1500 g	2(1.47)	45(1.89)			1(0.25)		
1500–2499 g	15(11.03)	366(15.41)			58(14.61)		
2500-4500 g	119(87.50)	1949(82.06)			334(84.13)		
> 4500 g	0(0.00)	15(0.63)			4(1.01)		
Low birth weight, n(%)	17(12.50)	411(17.31)	0.147	0.683(0.406-1.147)	59(14.86)	0.497	0.818(0.459-1.460)
Early neonatal death, n(%)	0(0.00)	4(0.17)	1.000	0.946(0.937-0.955)	0(0.00)	–	–
Congenital malformations, n(%)	3(2.21)	55(2.32)	1.000	0.951(0.294-3.081)	13(3.27)	0.772	0.666(0.187-2.374)
Singletons	3	31			7		
Multiples	0	24			6		

PF-TESA ICSI, ICSI using PF triggered frozen-thawed testicular spermatozoa; non-PF conventional ICSI, ICSI using fresh ejaculation without PF trigger.

Concerning the occurrence of birth defects, we observed 57 newborns with congenital malformations in the control group, whilst 3 out of 136 babies in the PF-TESA ICSI group were found. However, neither before nor after matching did we find any significant differences in the defect rate among the three groups (*P*>0.05). **Table 6** displayed the various types of congenital malformations observed in live-born infants.

**Table 6:** Types of congenital malformations among live-born infants.

Type of malformation	The code and diagnosis according the International Classification of Diseases, 10th Revision (n)	PF-TESA ICSI group	non-PF conventional ICSI group	non-PF TESA ICSI group
Circulatory system	Q21.0: Ventricular septal defect (5)	0	5	0
	Q21.102: atrial septal defect (10)	0	10	0
	Q21.103: patent foramen ovale (3)	0	3	0
	Q21.3: tetralogy of Fallot (1)	0	1	1
	Q25.0: patent ductus arteriosus (4)	0	4	0
	Q25.6: Stenosis of pulmonary artery (1)	0	1	0
	Q24.9 Congenital malformation of heart, unspecified (4)	0	4	0
	Q28.3: hemangioma (10)	1	9	0
Respiratory system	Q33.0 Congenital cystic lung (1)	0	1	0
Digestive system	K83.1: Obstruction of bile duct (1)	0	1	0
	Q38.1: ankyloglossia (1)	0	1	0
	Q43.9: Congenital malformation of intestine, unspecified (1)	0	1	0
	Q44.7: Other congenital malformations of liver (1)	0	1	0
musculoskeletal system	Q66.2: Metatarsus varus (1)	0	1	0
	Q69.9: polydactyly (2)	1	1	0
	Q70.0: Fused fingers (2)	0	2	0
	Q77.3: Chondrodysplasia punctata (2)	0	2	0
	Q79.8: Poland syndrome (1)	0	1	0
genitourinary system	N13.3: Other and unspecified hydronephrosis (1)	0	1	0
	N43.3: hydrocele (1)	1	0	0
Other malformations	D55.0: favism (1)	0	1	1
	I52.8: Other heart disorders in other diseases classified elsewhere (1)	0	1	0
	L91.8: Other hypertrophic disorders of skin (1)	0	1	0
	Q10.0:Congenital ptosis (1)	0	1	0
	Q13.4: Other congenital corneal malformations (1)	0	1	0
Total birth defects	2.33(60/2572)	3	55	2

PF-TESA ICSI, ICSI using PF triggered frozen-thawed testicular spermatozoa; non-PF TESA ICSI, ICSI using frozen-thawed testicular spermatozoa; non-PF conventional ICSI, ICSI using fresh ejaculation.

In addition, to ensure the stability of our research results, we conducted a subgroup analysis on patients who underwent the double cleavage embryo transfer, yielding results consistent with the primary analysis (**Supplementary Tables 1**-**5**).

## 4 Discussions

This study showed that ICSI using frozen-thawed testicular spermatozoa stimulated by PF was capable of achieving comparable implantation rates, clinical pregnancy rates, and live birth rates compared to the non-PF TESA ICSI and non-PF conventional ICSI groups. Moreover, there were no discernible differences in outcomes related to birth weight, gestational age or congenital defects delivery, comparing these findings to the non-PF TESA ICSI and non-PF conventional ICSI groups, too. This research provides the clinicians with conclusive evidences suggesting that the PF-TESA ICSI treatment might represent a more efficient and prompt method for the identification and selection of viable spermatozoa when performing ICSI using frozen-thawed TESA spermatozoa.

The effectiveness of PF on ICSI outcomes has been investigated in previous studies. One retrospective analysis of 47 ICSI cycles that used immotile testicular sperm treated with PF yielded a markedly higher fertilization rate, comparing to 30 ICSI cycles that utilized unselected immotile sperm, whilst other outcomes were comparable (18). Two prospective studies found that ICSI cycles using PF-treated immotile spermatozoa had similar clinical pregnancy outcomes compared to ICSI cycles using spontaneously motile spermatozoa (16, 17), which was similar with our results. Furthermore, while the hypo-osmotic swelling test selection is also usually utilized in TESA-recovered spermatozoa for ICSI, it is not fitting for spermatozoa that have been cryopreserved and thawed, as these cells spontaneously develop tail swelling (31). On the other hand, PF treatment does not contain this shortcoming. The use of viable spermatozoa acquired through PF was proved to be more effective in terms of fertilization and pregnancy outcomes compared to those obtained through the hypo-osmotic swelling test (19). However, a limitation of these studies is that none of them focused on neonatal outcomes, perhaps due to small sample sizes.

Currently, there is a dearth of data on babies born as a result of PF treatment. Navas et al. conducted a study of 102 patients who achieved clinical pregnancy after PF-ICSI and found that PF did not increase the risk of adverse obstetric and neonatal outcomes, including malformation rates (22). However, the study had some limitations, including a small cohort, heterogeneous sperm source (epididymal and testicular) and type (fresh and frozen-thawed), and an inadequate control group.

In our study, we compared the outcomes of PF-triggered frozen-thawed testicular spermatozoa, untreated testicular spermatozoa, and fresh spermatozoa in a larger cohort of patients. We found no significant differences in the pregnancy (**Tables 2**, **3**) and neonatal (**Tables 4**–**6**) outcomes among the three groups. Our findings suggest that the application of PF is unlikely to have adverse effects on pregnancy and neonatal outcomes in TESA patients undergoing PF-TESA ICSI treatment, which is consistent with the Navas et al.’s research (22).

Testicular spermatozoa are often immotile immediately after biopsy, especially after thawing of frozen samples (32). Motile spermatozoa, which exhibit reliable signs of vitality, are preferred for ICSI due to their significant impact on the procedure’s efficacy (9, 33). However, selecting viable testicular sperm for ICSI can be a challenging and lengthy process, particularly from frozen-thawed samples. In the study on ICSI with frozen-thawed testicular spermatozoa, Verheyen et al. reported that an average of 17 minutes was required to find motile spermatozoa (34). Another study (17) found that 21.3% of cycles had no motile spermatozoa after thawing, and in 38.0% of ICSI cycles, it took over 20 minutes to find motile spermatozoa per spermatozoon. The use of PF could initiate or stimulate the motility of spermatozoa when no motile spermatozoa were observed, significantly reducing the search time per spermatozoon. PF treatment facilitates the identification and selection of vital sperm for ICSI, and reduced operation time for staff involved in TESA (18). In this study, the mean duration of searching for a viable sperm in PF-TESA ICSI cycles was approximately 3.36 minutes (**Supplementary Table 6**), which is consistent with published study (18) (about 4.25 minutes for a viable sperm). Our study demonstrated that the use of PF not only reduced the time for operators to search for available spermatozoa during ICSI, but also provided embryologists with more opportunities to select spermatozoa with optimal morphology from a larger pool of motile spermatozoa. This ensured that ICSI procedures were conducted using morphologically normal and motile sperm, thereby averting potential adverse effects caused by the selection of immotile or morphologically abnormal sperm. Furthermore, the use of PF indirectly or even directly mitigated the risk of encountering insufficient available spermatozoa in frozen-thawed TESA-ICSI cycles, consequently reducing the likelihood of secondary testicular puncture in patients and alleviating their financial burden. In a word, selecting motile spermatozoa for ICSI is essential, and PF treatment can streamline the process, resulting in comparable outcomes for the procedure, especially for frozen-thawing testicular spermatozoa.

Despite the evident clinical advantages of PF application, its potential embryotoxicity based on animal studies should draw our attention. Previous research conducted on mice embryos revealed that although no differences were noted in the early development of 1-cell embryos cultured in micromolar concentration of PF, exposure to millimolar concentrations of PF resulted in the blocking of 2-cell embryos, reduced cell numbers of blastocysts, and impaired implantation and egg-cylinder formation (20, 21). The toxic effects found in animal studies may be caused by direct contact of embryos with PF. In a clinical setting, PF was only present in the prepared sperm sample, and the eggs/embryos were not exposed to culture drops (35), which was consistent with our study. It has been demonstrated that the embryotoxicity associated with PF was not observed in a sizable human population due to the sperm-washing protocol that mitigated potential harmful effects on post-fertilization development (36, 37). In our study, motile spermatozoa were selected and mechanically washed in the polyvinylpyrrolidone droplet using an injection pipette after PF exposure, to further minimize the risk of injecting PF into the oocyte during ICSI, similar to the previous report (17). Although our study did not show an increased risk of offspring associated with the use of PF to select mobile spermatozoa in ICSI patients using frozen-thawed testicular spermatozoa, its usage should be restricted to selected cases rather than being used indiscriminately.

Our study showed comparable pregnancy and neonatal outcomes between the TESA-ICSI cycle using frozen-thawed spermatozoa and the conventional ICSI cycle using fresh ejaculation. The similarity may be attributed to our consistent selection of motile and morphologically normal sperm by embryologists for ICSI, thereby mitigating potential adverse effects associated with the use of immotile or morphologically abnormal sperm. Moreover, only good-quality embryos were frozen for subsequent FET. The results suggest that embryos fertilized with motile spermatozoa exhibiting normal morphology in frozen-thawed TESA-ICSI procedures can achieve pregnancy and neonatal outcomes similar as those from conventional ICSI cycles using fresh spermatozoa, provided they develop into good-quality embryos.

This study included 240 patients who received PF-TESA ICSI treatment, making it the largest study on this topic to date and offering an opportunity to make a comprehensive comparison of neonatal outcomes among the PF-TESA ICSI, non-PF TESA ICSI and the conventional ICSI using fresh ejaculation. Additionally, in order to ensure the similarity of patient characteristics across the three groups, we employed the propensity score matching method, which allowed us to control for patient characteristics and make outcomes under the matched groups independent of treatment assignmen (38). Furthermore, we collected data only from patients with semen source of frozen-thawed TESA and fresh ejaculation in our center to minimize the influence of semen source on the outcomes.

This study had several limitations that should be acknowledged. Firstly, the analysis only assessed short neonatal outcomes, thereby the safety of PF remains a concern in ART. It is paramount to elucidate if PF gives rise to any safety issues and ascertain the optimal protocol, agent, dose, and duration of exposure during long-term follow-up studies (39). Secondly, as a retrospective study from a single center, a well-designed, prospective, randomized study in patients utilizing PF-TESA ICSI should be conducted in the future. Thirdly, the lack of information concerning birth defects among women experiencing miscarriage related to fetal abnormality where in defects may have been present could lead to the underestimation of the true prevalence of birth defects (40).

## 5 Conclusions

The PF-TESA ICSI methodology demonstrates comparable pregnancy and neonatal outcomes, which will be beneficial to patients using frozen-thawed testicular spermatozoa.

## Data availability statement

The original contributions presented in the study are included in the article/**Supplementary Material**. Further inquiries can be directed to the corresponding author.

## Ethics statement

The studies involving humans were approved by the Ethics Committee of Shanghai Ninth People’s Hospital, affiliated to Shanghai Jiao Tong University School of Medicine. The studies were conducted in accordance with the local legislation and institutional requirements. Written informed consent for participation was not required from the participants or the participants’ legal guardians/next of kin in accordance with the national legislation and institutional requirements.

## Author contributions

JD: Writing – review & editing, Writing – original draft, Software, Methodology, Formal analysis, Data curation. MY: Writing – review & editing, Writing – original draft, Software, Methodology, Formal analysis, Data curation. LW: Writing – original draft, Resources, Data curation. TW: Writing – original draft, Resources, Data curation. ML: Writing – review & editing, Resources, Data curation. WZ: Writing – review & editing, Resources, Conceptualization. MM: Writing – review & editing, Resources, Conceptualization. BL: Writing – review & editing, Visualization, Validation, Supervision, Investigation, Funding acquisition, Formal analysis, Conceptualization.
