# Supplementary material for: Pregnancy and neonatal outcomes of ICSI using pentoxifylline to identify viable spermatozoa in patients with frozen-thawed testicular spermatozoa
Source: Front Endocrinol (Lausanne). 2024 May 15;15:1364285. doi: 10.3389/fendo.2024.1364285 (PMC11133548; doi:10.3389/fendo.2024.1364285)
Supplement: Supplementary file 1 [file Table_1.docx]

| Supplemental Table 1. Baseline characteristics of patients who underwent double cleavage embryos transfer among the PF-TESA ICSI, non-PF TESA ICSI and non-PF conventional ICSI groups | | | | | | | | | | |
| --- | --- | --- | --- | --- | --- | --- | --- | --- | --- | --- |
| Characteristics | Study group | PF-TESA ICSI vs. non-PF conventional ICSI | | | | PF-TESA ICSI vs. non-PF TESA ICSI | | | | |
|  |  | Before matching | | After matching | | Before matching | | After matching | | |
|  |  | Control group 2 | *P* value | Control group 2 | *P* value | Control group 1 | *P* value | Study group | Control group 1 | *P* value |
| No. of patients | 150 | 3475 |  | 441 |  | 93 |  | 87 | 87 |  |
| Female age, years | 34(24-49) | 37(25-57) | **<0.001** | 35(25-53) | 0.984 | 37(30-58) | **<0.001** | 36(27-49) | 36(30-52) | 0.477 |
| Male age, years | 36.5(28-65) | 39(25-69) | **0.002** | 37(27-68) | 0.835 | 39(31-64) | **0.007** | 38(28-65) | 38(31-62) | 0.236 |
| Female body mass index, kg/m^2^ | 21.09(15.63-32.39) | 21.48(14.53-46.88) | **0.015** | 21.19(15.05-39.06) | 0.548 | 21.08(15.82-30.11) | 0.777 | 21.23(16.73-32.39) | 21.08(15.82-30.11) | 0.765 |
| Male body mass index, kg/m^2^ | 24.00(17.30-34.66) | 24.22(16.14-54.35) | 0.180 | 24.22(16.41-38.82) | 0.360 | 24.14(15.36-35.49) | 0.848 | 24.03(17.30-34.66) | 24.14(15.36-35.49) | 0.750 |
| Duration of infertility, years | 3(1-15) | 3(1-23) | **0.015** | 3(1-14)） | 0.541 | 3(0.5-17) | 0.720 | 3(1-15) | 3(0.5-14) | 0.521 |
| Gravidity | 0(0-5) | 0(0-9) | **<0.001** | 0(0-4) | 0.544 | 0(0-5) | 0.057 | 0(0-3) | 0(0-3) | 0.131 |
| Parity | 0(0-2) | 0(0-3) | 0.809 | 0(0-2) | 0.360 | 0(0-1) | 0.881 | 0(0-2) | 0(0-1) | 0.771 |
| No. of previous full-term births | 0(0-2) | 0(0-2) | 0.623 | 0(0-2) | 0.667 | 0(0-1) | 0.733 | 0(0-2) | 0(0-1) | 0.982 |
| No. of previous transfer failure cycles | 0(0-4) | 0(0-11) | **<0.001** | 0(0-4) | 0.366 | 0(0-4) | 0.319 | 0(0-4) | 0(0-4) | 0.975 |
| No. of previous no available embryo cycles | 0(0-2) | 0(0-8) | **0.007** | 0(0-4) | 0.768 | 0(0-2) | **0.011** | 0(0-2) | 0(0-2) | 0.794 |
| Causes of infertility |  |  |  |  |  |  |  |  |  |  |
| Tubal factor infertility, n (%) | 19(12.67) | 1775(51.08) | **<0.001** | 74(16.78) | 0.232 | 13(13.98) | 0.846 | 11(12.64) | 10(11.49) | 0.816 |
| Polycystic ovary syndrome, n (%) | 7(4.67) | 303(8.72) | 0.082 | 19(4.31) | 0.853 | 2(2.15) | 0.489 | 2(2.30) | 2(2.30) | 1.000 |
| Endometriosis, n (%) | 2(1.33) | 324(9.32) | **0.001** | 4(0.91) | 0.647 | 4(4.30) | 0.206 | 1(1.15) | 4(4.60) | 0.368 |
| Male factor infertility, n (%) | 150(100.00) | 1527(43.94) | **<0.001** | 441(100.00) | - | 93(100.00) | - |  |  |  |
| Endometrial thickness, mm | 10.45(5.10-22.80) | 10.40(3.70-22.80) | 0.404 |  | 0.589 | 10.70(6.80-21.70) | 0.894 | 10.50(5.10-17.30） |  | 0.678 |
| Endometrium preparation |  |  | 0.154 |  | 0.984 |  | 0.208 |  |  | 0.314 |
| Natural cycles, n (%) | 35(23.33) | 657(18.91) |  | 106(24.04) |  | 24(25.81) |  | 22(25.29) | 21(24.14) |  |
| Stimulated cycles, n (%) | 87(58.00) | 1957(56.32) |  | 254(57.60) |  | 44(47.31) |  | 51(58.62) | 44(50.57) |  |
| Hormone therapy cycles, n (%) | 28(18.67) | 861(24.78) |  | 81(18.37) |  | 25(26.88) |  | 14(16.09) | 22(25.29) |  |
| PF-TESA ICSI, ICSI using PF triggered frozen-thawed testicular spermatozoa; non-PF TESA ICSI, ICSI using frozen-thawed testicular spermatozoa; non-PF conventional ICSI, ICSI using fresh ejaculation; Bold indicates *P* < 0.05. | | | | | | | | | | |
