# Supplementary material for: Pregnancy and neonatal outcomes of ICSI using pentoxifylline to identify viable spermatozoa in patients with frozen-thawed testicular spermatozoa
Source: Front Endocrinol (Lausanne). 2024 May 15;15:1364285. doi: 10.3389/fendo.2024.1364285 (PMC11133548; doi:10.3389/fendo.2024.1364285)
Supplement: Supplementary file 2 [file Table_2.docx]

| Supplemental Table 2. Pregnancy outcomes of patients who underwent double cleavage embryos transfer between the PF-TESA ICSI and non-PF TESA ICSI groups | | | | | | | |  |
| --- | --- | --- | --- | --- | --- | --- | --- | --- |
|  |  | PF-TESA ICSI (study group) vs. non-PF TESA ICSI (control group 1) | | | | | | |
| Outcomes | Before matching | | | | After matching | | | |
|  | Study group | Control group 1 | *P* value | OR(95%CI) | Study group | Control group 1 | *P* value | OR(95%CI) |
| Biochemical pregnancy | 63.33(95/150) | 59.14(55/93) | 0.513 | 1.193(0.702-2.028) | 58.62(51/87) | 62.07(54/87) | 0.642 | 0.866(0.471-1.590) |
| Clinical pregnancy | 60.00(90/150) | 53.76(50/93) | 0.339 | 1.290(0.765-2.175) | 55.17(48/87) | 57.47(50/87) | 0.760 | 0.911(0.500-1.658) |
| Intrauterine implantation | 38.33(115/300) | 34.95(65/186) | 0.452 | 1.157(0.791-1.693) | 34.48(60/174) | 37.36(65/174) | 0.576 | 0.883(0.569-1.368) |
| Ectopic pregnancy | 1.11(1/90) | 4.00(2/50) | 0.290 | 0.270(0.024-3.051) | 2.08(1/48) | 4.00(2/50) | 1.000 | 0.511(0.045-5.823） |
| Multiple gestation | 23.33(21/90) | 28.00(14/50) | 0.541 | 0.783(0.356-1.720) | 25.00(12/48) | 28.00(14/500 | 0.737 | 0.857(0.349-2.106) |
| Miscarriage | 12.22(11/90) | 6.00(3/50) | 0.240 | 2.181(0.579-8.221) | 4.17(2/48) | 6.00(3/50) | 1.000 | 0.681(0.109-4.267) |
| Live birth | 52.00(78/150) | 48.39(45/93) | 0.584 | 1.156(0.689-1.939) | 51.72(45/87) | 51.72(45/87) | 1.000 | 1.000(0.552-1.812) |
| Multiple birth | 25.64(20/78) | 24.44(11/45) | 0.883 | 1.066(0.456-2.491) | 26.67(12/45) | 24.44(11/45) | 0.809 | 1.124(0.436-2.900) |
| Gestational age (weeks,mean (SD)) | 39(26-42) | 38(24-41) | 0.552 | - | 38(24-410 | 38（34-41） | 0.931 | - |
| Gestational age (weeks,n(%)) |  |  | 0.242 | - |  |  | 0.353 | - |
| < 32 | 1(1.28) | 1(2.22) |  |  | 0(0.00) | 1(2.22) |  |  |
| 32 - 36 | 6(7.69) | 9(20.00) |  |  | 5(11.11) | 9(20.00) |  |  |
| 37 - 40 | 66(84.62) | 32(71.11) |  |  | 38(84.44) | 32(71.11) |  |  |
| > 42 | 5(6.41) | 3(6.67) |  |  | 2(4.44) | 3(6.67) |  |  |
| Preterm delivery (<37 weeks)(%) | 8.97(7/78) | 22.22(10/45) | **0.040** | **0.345(0.121-0.983)** | 11.11(5/45) | 22.22(10/45) | 0.157 | 0.438(0.136-1.403) |
| PF-TESA ICSI, ICSI using PF triggered frozen-thawed testicular spermatozoa; non-PF TESA ICSI, ICSI using frozen-thawed testicular spermatozoa; Bold indicates *P* < 0.05. | | | | | | | | |
