# Supplementary material for: Pregnancy and neonatal outcomes of ICSI using pentoxifylline to identify viable spermatozoa in patients with frozen-thawed testicular spermatozoa
Source: Front Endocrinol (Lausanne). 2024 May 15;15:1364285. doi: 10.3389/fendo.2024.1364285 (PMC11133548; doi:10.3389/fendo.2024.1364285)
Supplement: Supplementary file 3 [file Table_3.docx]

| Supplemental Table 3. Pregnancy outcomes of patients who underwent double cleavage embryos transfer between the PF-TESA ICSI and non-PF conventional ICSI groups | | | | | | | |
| --- | --- | --- | --- | --- | --- | --- | --- |
| Outcomes | PF-TESA ICSI (study group) versus non-PF conventional ICSI (control group 2) | | | | | | |
|  | Study group | Before matching | | | After matching | | |
|  |  | Control group 2 | *P* value | OR(95%CI) | Control group 2 | *P* value | OR(95%CI) |
| Biochemical pregnancy | 63.33(95/150) | 52.95(1840/3475) | **0.013** | **1.535(-1.094-2.154)** | 65.76(290/441) | 0.590 | 0.899(0.611-1.323) |
| Clinical pregnancy | 60.00(90/150) | 49.29(1713/3475) | **0.010** | **1.543(1.106-2.153)** | 62.36(275/441) | 0.608 | 0.905(0.620-1.323) |
| Intrauterine implantation | 38.33(115/300) | 31.25(2172/6950) | **0.010** | **1.367(1.078-1.735)** | 39.80(351/882) | 0.654 | 0.940(0.719-1.231) |
| Ectopic pregnancy | 1.11(1/90) | 2.22(38/1713) | 0.719 | 0.495(0.067-3.649) | 0.36(1/275) | 0.433 | 3.079(0.191-49.729) |
| Multiple gestation | 23.33(21/90) | 24.81(425/1713) | 0.752 | 0.922(0.559-1.522) | 23.27(64/275) | 0.991 | 1.003(0.572-1.762) |
| Miscarriage | 12.22(11/90) | 13.72(235/1713) | 0.687 | 0.876(0.459-1.670) | 13.09(36/275) | 0.831 | 0.924(0.449-1.902) |
| Live birth | 52.00(78/150) | 41.58(1445/3475) | **0.011** | **1.522(1.097-2.111)** | 53.97(238/441) | 0.676 | 0.924(0.638-1.339) |
| Multiple birth | 25.64(20/78) | 26.23(379/1445) | 0.909 | 0.970(0.576-1.634) | 23.95(57/238) | 0.763 | 1.095(0.608-1.973) |
| Gestational age (weeks) | 39(26-42) | 38(25-42) | 0.172 | - | 39(31-41) | 0.911 | - |
| Gestational age (weeks, n(%)) |  |  | 0.198 | - |  | 0.724 | - |
| < 32 | 1(1.28) | 33(2.28) |  |  | 2(0.84) |  |  |
| 32 - 36 | 6(7.69) | 201(13.91) |  |  | 28(11.76) |  |  |
| 37 - 40 | 66(84.62) | 1163(80.48) |  |  | 196(82.35) |  |  |
| > 42 | 5(6.41) | 48(3.32) |  |  | 12(5.04) |  |  |
| Preterm delivery (<37 weeks)(%) | 8.97(7/78) | 15.78(228/1445) | 0.105 | 0.526(0.239-1.159) | 12.61(30/238) | 0.387 | 0.684(0.288-1.625) |
| PF-TESA ICSI, ICSI using PF triggered frozen-thawed testicular spermatozoa; non-PF conventional ICSI, ICSI using fresh ejaculation without PF trigger; Bold indicates *P* < 0.05. | | | | | | | |
