# Supplementary material for: Pregnancy and neonatal outcomes of ICSI using pentoxifylline to identify viable spermatozoa in patients with frozen-thawed testicular spermatozoa
Source: Front Endocrinol (Lausanne). 2024 May 15;15:1364285. doi: 10.3389/fendo.2024.1364285 (PMC11133548; doi:10.3389/fendo.2024.1364285)
Supplement: Supplementary file 4 [file Table_4.docx]

| Supplemental Table 4. Neonatal outcomes of patients who underwent double cleavage embryos transfer between the PF-TESA ICSI and non-PF TESA ICSI groups | | | | | | | |  |
| --- | --- | --- | --- | --- | --- | --- | --- | --- |
| Outcomes |  | PF-TESA ICSI (study group) vs. non-PF TESA ICSI (control group 1) | | | | | | |
|  | Before matching | | | | After matching | | | |
|  | Study group | Control group 1 | *P* value | OR(95%CI) | Study group | Control group 1 | *P* value | OR(95%CI) |
| Live born infants (*n*) | 98 | 56 | 0.852 | 1.066(0.545-2.084) | 57 | 56 | 0.760 | 1.124(0.530-2.382) |
| Single | 58(59.18) | 34(60.71) |  |  | 33(57.89) | 34(60.71) |  |  |
| Twins | 40(40.82) | 22(39.29) |  |  | 24(42.11) | 22(39.29) |  |  |
| Birthweight (g) | 3000(995-4400) | 3000(2000-4250) | 0.416 | - | 3000(1800-4400) | 3000(2000-4250) | 0.302 | - |
| Birthweight, (g, n(% )) |  |  | 0.627 | - |  |  | 0.616 | - |
| < 1500 g | 1(1.02) | 0(0.00) |  |  | 0(0.00) | 0(0.00) |  |  |
| 1500–2499 g | 13(13.27) | 7(12.50) |  |  | 9(15.79) | 7(12.50) |  |  |
| 2500-4500 g | 84(85.71) | 49(87.50) |  |  | 48(84.21) | 49(87.50) |  |  |
| > 4500 g | 0(0.00) | 0(0.00) |  |  | 0(0.00) | 0(0.00) | - | - |
| Low birth weight, n(% ) | 14(14.29) | 7(12.50) | 0.756 | 1.167(0.441-3.088) | 9(15.79) | 7(12.50) | 0.616 | 1.313(0.452-3.807) |
| Early neonatal death, n(% ) | 0(0.00) | 0(0.00) | - | - | 0(0.00) | 0(0.00) | - | - |
| Congenital malformations, n(% ) | 3(3.06) | 2(3.57) | 1.000 | 0.853(0.138-5.263) | 1(1.75) | 2(3.57) | 0.618 | 0.482(0.042-5.473) |
| Singletons | 3 | 1 |  |  | 1 | 1 |  |  |
| Multiples | 0 | 1 |  |  | 0 | 1 |  |  |
| PF-TESA ICSI, ICSI using PF triggered frozen-thawed testicular spermatozoa; non-PF TESA ICSI, ICSI using frozen-thawed testicular spermatozoa | | | | | | |  |  |
