# Supplementary material for: Pregnancy and neonatal outcomes of ICSI using pentoxifylline to identify viable spermatozoa in patients with frozen-thawed testicular spermatozoa
Source: Front Endocrinol (Lausanne). 2024 May 15;15:1364285. doi: 10.3389/fendo.2024.1364285 (PMC11133548; doi:10.3389/fendo.2024.1364285)
Supplement: Supplementary file 5 [file Table_5.docx]

| Supplemental Table 5. Neonatal outcomes of patients who underwent double cleavage embryos transfer between the PF-TESA ICSI and non-PF conventional ICSI groups | | | | | | | |
| --- | --- | --- | --- | --- | --- | --- | --- |
| Outcomes | PF-TESA ICSI (study group) versus conventional ICSI (control group 2) | | | | | | |
|  | Study group | Before matching | | | After matching | | |
|  |  | Control group 2 | *P* value | OR(95%CI) | Control group 2 | *P* value | OR(95%CI) |
| Live born infants (*n*) | 98 | 1822 | 0.895 | 0.972(0.643-1.470) | 294 | 0.676 | 1.105(0.693-1.761) |
| Single | 58(59.18) | 1066(58.51) |  |  | 181(61.56) |  |  |
| Twins | 40(40.82) | 756(41.49) |  |  | 113(38.44) |  |  |
| Birthweight (g) | 3000(995-4400) | 3000(690-5300) | 0.899 | - | 3035(1800-4800) | 0.483 | - |
| Birthweight, (g, n(% )) |  |  | 0.408 | - |  | 0.111 | - |
| < 1500 g | 1(1.02) | 40(2.20) |  |  | 0(0.00) |  |  |
| 1500–2499 g | 14(14.29) | 324(17.78) |  |  | 58(19.73) |  |  |
| 2500-4500 g | 83(84.69) | 1447(79.42) |  |  | 233(79.25) |  |  |
| > 4500 g | 0(0.00) | 11(0.60) |  |  | 3(1.02) |  |  |
| Low birth weight, n(% ) | 15(15.31) | 364(19.98) | 0.258 | 0.724(0.413-1.270) | 58(19.73) | 0.330 | 0.735(0.395-1.368) |
| Early neonatal death, n(% ) | 0(0.00) | 4(0.22) | 1.000 | 0.949(0.939-0.959) | 0(0.00) |  |  |
| Congenital malformations, n(% ) | 3(3.06) | 45(2.47) | 0.734 | 1.247(0.381-4.086) | 9(3.06) | 1.000 | 1.000(0.265-3.770) |
| Singletons | 3 | 21 |  |  | 6 |  |  |
| Multiples | 0 | 24 |  |  | 3 |  |  |
| PF-TESA ICSI, ICSI using PF triggered frozen-thawed testicular spermatozoa; non-PF conventional ICSI, ICSI using fresh ejaculation without PF trigger | | | | | | | |
