# Supplementary material for: Pregnancy and neonatal outcomes of ICSI using pentoxifylline to identify viable spermatozoa in patients with frozen-thawed testicular spermatozoa
Source: Front Endocrinol (Lausanne). 2024 May 15;15:1364285. doi: 10.3389/fendo.2024.1364285 (PMC11133548; doi:10.3389/fendo.2024.1364285)
Supplement: Supplementary file 6 [file Table_6.docx]

| Supplemental Table 6. Mean time for sperm searching in thawed TESA-ICSI cycles using PF treatment | |
| --- | --- |
| No. of cycles | 18 |
| No. of metaphase II oocytes | 94 |
| No. of fertilized (2PN) oocytes | 81 |
| No. of total fertilization failure cycles (%) | 0 |
| Normal fertilization rate/metaphase II oocyte (%) | 81/94(86.17) |
| Good quality embryo rate/fertilized (2PN) oocyte (%) | 44/81(54.32) |
| Blastocyst formation rate (%) | 7/34(20.59) |
| Egg utilization rate (%) | 48/137(35.04) |
| No. of embryos, median(min–max) | 2(0-7) |
| Mean time for sperm searching, isolation and ICSI per cycle, minutes (min–max) | 17.53(1.5-30) |
| Mean time for sperm searching, isolation and ICSI per oocyte, minutes (min–max) | 3.36(315.48/94) |
| TESA, testicular sperm aspiration; ICSI, intracytoplasmic sperm injection; PF, pentoxifylline | |
